# IMGT/GeneInfo: T cell receptor gamma TRG and delta TRD genes in database give access to all TR potential V(D)J recombinations

**DOI:** 10.1186/1471-2105-7-224

**Published:** 2006-04-26

**Authors:** Thierry-Pascal Baum, Vivien Hierle, Nicolas Pasqual, Fatena Bellahcene, Denys Chaume, Marie-Paule Lefranc, Evelyne Jouvin-Marche, Patrice Noël Marche, Jacques Demongeot

**Affiliations:** 1 Laboratoire TIMC-IMAG-CNRS UMR 5525, Techniques de l'Imagerie, de la Modélisation et de la Cognition; Université Joseph Fourier, Grenoble 1, Faculté de Médecine, Domaine de la Merci, 38706 La Tronche, France; 2 Laboratoire d'Immunochimie, CEA-Grenoble/DRDC/ICH; INSERM U548; Université Joseph Fourier Grenoble 1, 17 rue des Martyrs, 38054 Grenoble Cedex 09, France; 3 Laboratoire d'ImmunoGénétique Moléculaire, LIGM; Université Montpellier II, UPR CNRS 1142, IGH, 141 rue de la Cardonille, 34396 Montpellier Cedex 5, France; 4 Institut Universitaire de France, 103 Boulevard Saint-Michel, 75005 Paris, France; 5 ImmunID Technologies, 17 rue des Martyrs, 38054 Grenoble Cedex 09, France

## Abstract

**Background:**

Adaptative immune repertoire diversity in vertebrate species is generated by recombination of variable (V), diversity (D) and joining (J) genes in the immunoglobulin (IG) loci of B lymphocytes and in the T cell receptor (TR) loci of T lymphocytes. These V-J and V-D-J gene rearrangements at the DNA level involve recombination signal sequences (RSS). Whereas many data exist, they are scattered in non specialized resources with different nomenclatures (eg. flat files) and are difficult to extract.

**Description:**

IMGT/GeneInfo is an online information system that provides, through a user-friendly interface, exhaustive information resulting from the complex mechanisms of T cell receptor V-J and V-D-J recombinations. T cells comprise two populations which express the αβ and γδ TR, respectively. The first version of the system dealt with the *Homo sapiens *and *Mus musculus *TRA and TRB loci whose gene rearrangements allow the synthesis of the αβ TR chains. In this paper, we present the second version of IMGT/GeneInfo where we complete the database for the *Homo sapiens *and *Mus musculus *TRG and TRD loci along with the introduction of a quality control procedure for existing and new data. We also include new functionalities to the four loci analysis, giving, to date, a very informative tool which allows to work on V(D)J genes of all TR loci in both human and mouse species. IMGT/GeneInfo provides more than 59,000 rearrangement combinations with a full gene description which is freely available at .

**Conclusion:**

IMGT/GeneInfo allows all TR information sequences to be in the same spot, and are now available within two computer-mouse clicks. This is useful for biologists and bioinformaticians for the study of T lymphocyte V(D)J gene rearrangements and their applications in immune response analysis.

## Background

The development of a mature and diverse adaptive immune response in vertebrate species require DNA rearrangements of the variable (V), diversity (D) and joining (J) genes in the immunoglobulin (IG) loci of B lymphocytes, and in the T cell receptor (TR) loci of T lymphocytes [1,2]. Recombination is made by RAG1 and RAG2 enzymes which recognize the recombination signal sequences (RSSs) located at the borders of each V, D and J rearranging gene [3]. The RSS contains a conserved heptamer and nonamer, separated by a non-conserved spacer of 12 or 23 base pairs [1,2]. There are two populations of T lymphocytes, the αβ and the γδ T cells, depending on their receptors, αβ and γδ TR, respectively.

The IMGT/GeneInfo information system, a collaborative part of IMGT^®^, the international ImMunoGeneTics information system^® ^[4], is intended to give a user-friendly and intuitive access to data about the recombination of V, J and, if present, D genes, which are used to build the antigen receptors expressed by the lymphocytes. Compared to existing systems, IMGT/GeneInfo gathers and synthetizes information from publicly available databases, and offer this information in a unique place which is readily available within seconds through a simple drop-down boxes 2-step process. IG and TR are the antigen receptors of the B and T cells, respectively. The TR genes are located in four loci, designated as TRA, TRB, TRG and TRD which contribute to the synthesis of the alpha, beta, gamma and delta chains, respectively[2,5,6].

In the first version of IMGT/GeneInfo [7], we addressed TRA and TRB loci for *Homo sapiens *and *Mus musculus*. In this second version, we extend our information system in 3 ways by 1/adding new data on TRG and TRD loci, 2/introducing a quality control procedure designed to check existing and new data, and 3/implementing new functionalities designed to enhance user interface and interactivity, as well as giving a better integration with IMGT^® ^databases and tools.

The IMGT/GeneInfo extension will be helpful for immunologists to accurately describe TR gene rearrangements, in the course of T cell functions and development. Now, the new site includes the whole set of TR genes (TRA, TRB, TRG and TRD) for man and mouse species. The IMGT/GeneInfo Query page remains a two step process: the choice of species and chain on the first page and then the choice of genes on the second page. Results appear on the third page with 7 parts (two new parts). New features and enhancements both make the tool closer to user's needs and decrease error risks.

## Construction and content

### IMGT/GeneInfo new data

In order to construct the database, initial sequence information was retrieved from the publicly available databases. Data collection was made from three different data sources: annotation files (from EMBL/GenBank/DDBJ), IMGT/LIGM-DB annotated flat files [8], and the T cell receptor FactsBook [2]. Data extraction was carried out in two ways: first automatically by a dedicated C++ program, and then manually where results of the program were verified for concordance of gene description between the 3 sources before entering them into the database. Once in database, data have been again checked for consistency with the 3 sources to verify if our data collection is complete and free of errors when generated. Data verification has been made according to a quality control especially developed for that purpose (cf. infra).

In this second version, we are extending to the *Homo sapiens *and *Mus musculus *TRG and TRD loci whose gene rearrangements allow the synthesis of the two γ and δ chains of the TR [2,6]. Thus, we now have completed the two populations of TR cells.

Gamma-delta T cells are distinct from αβ T cells, by their functions, by the genes which encode their TR, by the nature of the recognized antigens, and by the type of antigen presenting receptors [9-11]. While αβ T cells recognize a broad range of target processed peptides presented by highly polymorphic classical MHC molecules [12-14], γδ T cells recognize pathogen glycolipids or phospholipids usually presented by non polymorphic receptors belonging to the MHC superfamily (MhcSF) such as CD1 [2,11]. Furthermore, γδ T cells are known to appear earlier than αβ T cells during the ontogeny in mouse and probably in human [15-17].

The human and mouse TRD locus, located on chromosome 14 in both species, is embedded within the TRA locus and shares several V genes with the TRA locus [2,9,10]. Moreover, the TRD locus has D genes, J genes and one constant C gene (Table 1). The human TRG locus, which is located on chromosome 7 at 7p14, comprises 14 V genes, 5 J genes and 2 C genes [2], whereas the mouse TRG locus, located on chromosome 13, is made of 7 V genes, 4 J genes and 4 C genes (IMGT Repertoire, ) (Table 1).

In order to apprehend and to be able to use all the complexity of these loci and their potential rearrangements, we have taken into account TR parameters within 5 main categories: 1) gene names in IMGT^® ^standardized nomenclature [18] with their correspondence in previous nomenclatures [19,20]; 2) the gene functionality (functional, open reading frame ORF or pseudogene) and other identification criteria (rearrangement, transcription); 3) RSS sequences, composed of an heptamer (V-HEPTAMER), a spacer (V-SPACER) and a nonamer (V-NONAMER); 4) gene sequences, which include, for example for a V gene, the exon1 (or L-PART1 which encodes the first part of the leader), the intron (or V-INTRON), the exon2 (or V-EXON which encodes L-PART2, second part of the leader, and the core V-REGION); 5) general information data such as the accession numbers for sources, gene positions and their relative distances (in base pairs) in the locus, and relevant consensus sequences.

We used the IMGT Scientific chart rules that are based on the IMGT-ONTOLOGY concepts [21] for standardized nomenclature, keywords, labels and sequence delimitations concerning genes and RSS. The sources were IMGT/LIGM-DB annotated files [8], designated by the same accession numbers as EMBL/GenBank/DDBJ (Table 1).

Overall, the total number of V(D)J combinations available for human and mouse TRG and TRD loci is 25,225 (details in Table 1).

### Implementation

Data organization has been made on the basis of a relational model. User access is made by a 3-tiers architecture: database is accessed by a web server through a Java servlet middleware. IMGT/GeneInfo is deployed in the IMGT^® ^information system using Java Servlet technology. The interface uses HTML, JavaScript and CSS.

### Quality control procedure

IMGT/GeneInfo provides more than 59,000 result pages for all TR loci. Each result page gives users a lot of information (comprising many sequences, etc.). We wanted to make sure that all these results were accurate and free of errors, and that concordance among data source files was correct. To reach that goal we built a quality control procedure to ensure the correctness and integrity of our data. This control was made available for data previously existing in the first version of the system (TRA and TRB genes), and also for the new data (TRG and TRD genes).

The quality control has been conducted in two steps: data source homogeneity verification and criteria check-list.

We used three different data sources: annotation files (from EMBL/GenBank/DDBJ), IMGT/LIGM-DB annotated flat files [8], and the T cell receptor FactsBook [2]. For each source, we verified the coherence of gene description.

Once data sources have been verified, we used them to check our information system according to the 8 following criteria: 1) Correspondence between various gene nomenclatures (IMGT Repertoire) in order to avoid confusions in gene names. 2) Locus order checking, giving the physical order of a gene in the locus and the total number of genes for each V(D)J locus to make sure that all TR genes are displayed. 3) Gene sequence delimitations (L-PART1, V-INTRON, V-EXON comprising L-PART2 and V-REGION, D-REGION and J-REGION). 4) RSS sequences (heptamer, nonamer and spacer). 5) Correspondence between DNA and "Spliced V-(D)-J rearranged sequence" parts. We verify in the results that these "spliced" sequences exactly correspond to the parts used to build them. 6) Assignment of a correct RSS position and gene direction for the V(D)J recombination. 7) Gene functionality checking (a germline V, D or J gene and a C gene can be functional, ORF or pseudogene). 8) Links validity: to ensure that each hypertext link opens the right Web page.

The procedure we used makes IMGT/GeneInfo a system with an exhaustive quality and gives us a rigorous traceability for each test.

## Utility and discussion

Immunologists working on repertoires are daily facing a huge amount of disseminated data usually hard to collect and to check. To work on TR, scientists must first select TR chain of interest which may include thousands of different forms. In IMGT/GeneInfo, we gather, connect and present all needed separated parameters in a unique place. Data are checked by a dedicated quality control. By adding TRG and TRD genes, we make available all TR information for mouse and human. This information is available and presented in a convenient way for users (biological researchers, bioinformaticians, etc.) through a simple and intuitive 2 steps enhanced interface.

**Figure 1:**
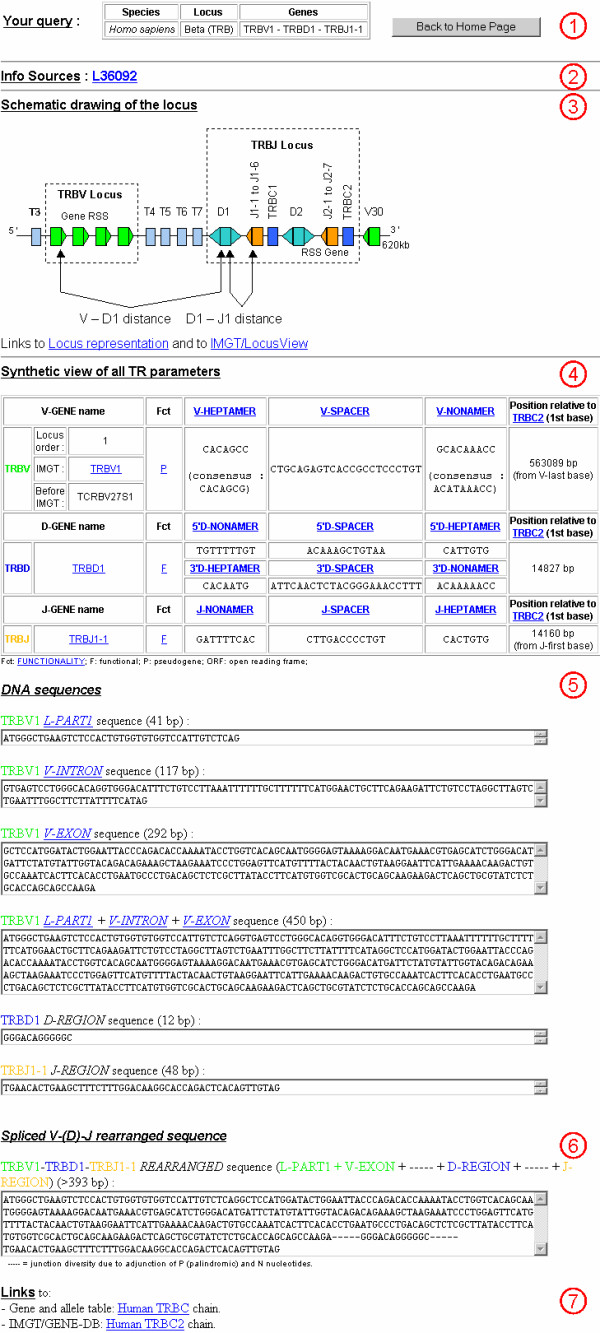
**IMGT/GeneInfo Results page**. Screen shot of IMGT/GeneInfo Results page. Result set for a query requesting a V-D-J recombination in human T cell receptor delta (TRD) locus with TRAV36/DV7, TRDD2 and TRDJ3 genes.

IMGT/GeneInfo is an information system, relying on an integrated relational database. It has been specifically designed to give users all information about V(D)J recombinations within two mouse-clicks. With the first click, the user selects the species, the locus and the type of gene combination (V-V, V-J, V-D-J) from a drop-down box [7]. With the second click, the user chooses within each type (V, D, J) the specific genes for which information is required. As opposed to other information systems, IMGT/GeneInfo does not take any user sequence for analysis. The list of genes available for user choice is determined from existing sequences from the major available databases (IMGT, EMBL, GenBank, and DDBJ). Gene choice can be made either according to the gene name, or the relative position of the gene within the locus. After the second click, user is directed to the results page. This page was enhanced in order to accelerate and make easier the rearrangement analysis (Figure 1). Now, each specific term is associated with its definition via a hypertext link. Results page is divided in seven parts (instead of 5 previously):  user query,  information sources,  schematic drawing of the locus,  synthetic view of all TR gene parameters,  DNA sequences,  spliced V-(D)-J rearranged sequences, and  link to the constant gene and allele tables in IMGT Repertoire. Among these 7 parts, the 2 new ones are:

***User query ***, which is a summary of the request according to species, locus and gene. This will facilitate printed data archiving;

***Spliced V-(D)-J rearranged sequence ***, which gives the transcribed sequence after rearrangement and splicing. Spliced V-(D)-J rearranged sequences are only provided for the TRA and TRG V-J rearrangements and for the TRB and TRD V-(D)-J rearrangements, excluding other odd combinations. These sequences display blunt ends of the rearranged V, D and J regions and are therefore useful for any comparison with *in vivo *rearranged sequences whose complex junctions result from nucleotide deletions and N-diversity nucleotide insertions [2]. More detailed analysis of the junctions can eventually be performed with IMGT/V-QUEST [22] and IMGT/JunctionAnalysis [23].

Besides the 2 new parts described above, we enhanced the 5 already existing parts in the results page.

***Info sources ***. As information sources, accession numbers are now linked to the IMGT/LIGM-DB [8] results page which provides IMGT annotations, IMGT flat file, FASTA format, EMBL flat file, coding regions with protein translation, sequence with 3 reading frames, dump format, IMGT/V-QUEST analysis.

***Schematic drawing of the locus ***. We created different pictures for each type of rearrangement in order to get a visual check of the user query. Two links have been added: the first one to IMGT Repertoire Locus representation, which is a complete locus representation compared to our schematic drawing, and the second one to the IMGT/LocusView tool which gives a graphical representation of the gene location inside the locus [24].

***Synthetic view of all TR gene parameters ***. Each label in the table follows the IMGT standardized labels (defined in the IMGT Scientific chart rules and based on the DESCRIPTION concept of IMGT-ONTOLOGY) [21]. Sequences of the labels of the inverted genes (human TRBV30 and TRDV3, mouse TRBV31, TRDV5, TRGV2 and TRGJ2) are presented in the "sense" DNA strand orientation 5'-3' (IMGT Index, DNA strand orientation, ). RSS labels are presented in the 5'-3' orientation. For each label, users can obtain the relevant definition. The gene name link connects to the IMGT/GENE-DB entry [18] which gives additional information such as the chromosomal localization, reference alleles, known cDNAs for a given gene, etc.

***DNA sequences ***. They are now headed by a title to differentiate them from the spliced V-(D)-J rearranged sequences. In addition, we provide the size of all sequences in base pairs. Besides the complete DNA sequence as usually given by other web sites, we provide the individual parts of the gene sequence (L-PART1, V-INTRON and V-EXON), for a convenient handling by biologists.

***Link ***. A link from the constant gene is directed to gene and allele table in IMGT Repertoire which describes the various alleles available for a given constant gene, and to IMGT/GENE-DB [18].

The advantage our system has over existing ones, is that all information concerning the TR is synthesized in one result page, and this page can be accessed in only two computer mouse-clicks. It is also designed in an educational way in that we have a drawing to explain how different parts are organized in the selected V(D)J rearrangement.

In existing applications, sequences are given in flat files (e.g. with line numbers and intergenic sequences).

Thus, the user must manually remove line numbers and then find where is the desired sequence before being able to extract it. In our system, the user can directly copy and paste the sequence without the risk of mistake during manual selection and extraction.

Advances in genomic and postgenomic technologies still need annotation of the genes and bioinformatics tools to select and analyze specific genes. IMGT/GeneInfo extension to the *Homo sapiens *and *Mus musculus *TRD and TRG loci will be helpful for users to gather pertinent information about antigen receptor genes which is required to accurately describe the αβ or γδ T cells. This integrated bioinformatics information system will allow a rapid and secure selection of all TR DNA sequences to study the rearrangement frequency by molecular approaches such as Southern blot, real time PCR, multiplex PCR or microarray assays.

### Future directions

Future work on IMGT/GeneInfo will proceed in two main directions. On the application side, we will provide a wider range of analysis tools aimed at evaluating and characterizing TR in terms of amino acid sequences. On the infrastructure side, we will increase the amount of data offered by IMGT/GeneInfo by integrating IG data. Finally, we are planning to extend IMGT/GeneInfo to include information about genes in other organisms besides human and mouse.

## Conclusion

IMGT/GeneInfo is an information system dedicated to immunogenetic sequences. It summarizes the publicly existing available information on human and mouse V(D)J recombinations. It is useful for scientists, especially molecular biologists and bioinformaticians and very easy to use since it requires only two clicks to access the results page. The main purpose of our information system is to integrate and provide, as a single and consistent resource, a large amount of data about this mechanism in a fast and convenient manner. This was accomplished by combining the information available in several public on-line resources, and creating a local database that is accessible through a Web-based front-end.

## Availability and requirements

IMGT/GeneInfo is publicly and freely available at the following address:  for academic research purpose. For any non academic use, please contact Thierry-Pascal BAUM at tpbaum@imag.fr.

Authors who use IMGT/GeneInfo are strongly encouraged to cite this article and the IMGT/GeneInfo home page URL, at .

## Authors' contributions

Authors participated to the different steps in the following way (alphabetical order): EJM, JD, MPL, PNM, and TPB conducted the premises and launch of the project. NP, TPB and VH made the conception. NP, TPB and VH realized the 2-step user design in accordance with the IMGT graphical chart for a fair integration of the site. Architecture, technical decisions and development were made by TPB and VH. DC and TPB carried out the application deployment. EJM, JD, MPL, PNM and NP gave their immunological expertise and reviewed data for their accuracy and coherence. NP, FB, TPB and VH carried out the quality control procedure. All authors participated in beta testing the site. Manuscript was drafted by TPB and VH and revised by all the authors. TPB assured the coordination of the project. All authors read and approved the final manuscript.

## Appendix – access and contact

IMGT/GeneInfo Homepage: 

IMGT/GeneInfo Contact: tpbaum@imag.fr

IMGT^® ^Homepage: 

IMGT^® ^Contact: lefranc@ligm.igh.cnrs.fr

TIMC Contact: tpbaum@imag.fr

ICH Contact: patrice.marche@cea.fr

LIGM Contact: lefranc@ligm.igh.cnrs.fr

## Figures and Tables

**Table 1:** Gamma and delta T cell receptor V-D-J genes. Human *Homo sapiens *and mouse *Mus musculus *T cell receptor gamma (TRG) and delta (TRD) variable (V), diversity (D), and joining (J) genes in IMGT/GeneInfo. Sources: IMGT/LIGM-DB [8,18] and EMBL/GenBank/DDBJ.

TR V(D)J loci	TRDV	TRAV	TRDD	TRDJ	TRAJ	TRGV	TRGJ
Human							
Chromosome	TRA/TRD: 14q11.2					TRG: 7p14	
Total no.	3	54	3	4	61	14	5
Functional	3	45	3	4	50	6	5
ORF		1			8	3	
Pseudogene		8			3	5	
Locus size (kb)	TRA/TRD: 850					TRG: 180	
Sources	TRA/TRD: AE000658-AE000662 and NG_001332					TRG: AF159056, X08084, M12950, M12960, M16016, M12961	
Mouse							
Chromosome	TRA/TRD: 14 (19.7 cM)					TRG: 13 (10.0 cM)	
Total no.	6	98	2	2	60	7	4
Functional	5	79	2	2	38	7	4
ORF		5			12		
Pseudogene	1	14			10		
Locus size (kb)	TRA/TRD: 1400					TRG: 200	
Sources	TRA/TRD: AE008683-AE008686					TRG: AF037352 and AF021335	
